# Last male sperm precedence is modulated by female remating rate in *Drosophila melanogaster*

**DOI:** 10.1002/evl3.50

**Published:** 2018-04-13

**Authors:** Meghan Laturney, Roel van Eijk, Jean‐Christophe Billeter

**Affiliations:** ^1^ Groningen Institute for Evolutionary Life Sciences University of Groningen PO Box 11103 Groningen 9700 CC The Netherlands

**Keywords:** Female reproductive behavior, last male sperm precedence, mating rate, polyandry, sperm storage

## Abstract

Following multiple matings, sperm from different males compete for fertilization within the female reproductive tract. In many species, this competition results in an unequal sharing of paternity that favors the most recent mate, termed last male sperm precedence (LMSP). Much of our understanding of LMSP comes from studies in *Drosophila melanogaster* that focus on twice‐mated females with standardized latencies between successive matings. Despite accumulating evidence indicating that females often mate with more than two males and exhibit variation in the latency between matings, the consequences of mating rate on LMSP are poorly understood. Here, we developed a paradigm utilizing *D. melanogaster* in which females remated at various time intervals with either two or three transgenic males that produce fluorescent sperm (green, red, or blue). This genetic manipulation enables paternity assessment of offspring and male‐specific sperm fate examination in female reproductive tracts. We found that remating latency had no relationship with LMSP in females that mated with two males. However, LMSP was significantly reduced in thrice‐mated females with short remating intervals; coinciding with reduced last‐male sperm storage. Thus, female remating rate influences the relative share of paternity, the overall clutch paternity diversity, and ultimately the acquisition of indirect genetic benefits to potentially maximize female reproductive success.

**Impact Summary**

Although females from most species mate with multiple males and produce offspring with varying paternity within the same clutch, little is known about the function of polyandry. As it is widespread, polyandry is assumed to be advantageous: females that accept several partners pass on more offspring and/or relatively successful offspring compared to monogamous females. However, exactly how taking on multiple mates results in higher female reproductive success remains unclear.

One explanation of polyandry is that by increasing the genetic diversity of their clutches, females increase the probability that a proportion of the offspring will have a well‐suited genetic combination for a future environment. Given that prospective conditions may be unpredictable, polyandrous females would optimize these chances by producing equal number of offspring from all mates. However, in many species paternity is biased in favor of the last male: a phenomenon known as last male sperm precedence. Although this outcome is advantageous to her most recent mate, it reduces her scope for benefits by reducing the potential offspring genetic diversity to that of a monogamous female.

We hypothesized that females can modulate the potency of LMSP by adjusting mating rate. By mating with many males and in quick succession, females may skew male–male sperm interactions, leading to a more equal share of paternity and thus greater clutch genetic diversity. To test this, we mated females with either two or three different males at varying remating intervals. Indeed, we found that thriced‐mated females who were quick to remate produced more balanced clutches. Female remating rate thus impacts the acquisition of indirect genetic benefits via the modulation of sperm competition. This suggests a mechanism through which polyandry can function to increase offspring genetic diversity.

The development of molecular techniques has enabled researchers to accurately assess paternity across taxa. Contrary to previous assumptions that females opt for a single partner, the paternal genetic diversity of offspring suggested that polyandry is an intrinsic element of female reproductive behavior for a wide range of animal groups (Birkhead and Møller 1998). Subsequent studies sought to understand why females frequently mate with multiple partners (reviewed by Gowaty 1994; Jennions and Petrie 2000; Simmons 2005; Parker and Birkhead 2013). One potential explanation of polyandry concerns the acquisition of indirect fitness benefits through which female reproductive success is enhanced by increasing the chances of survival/reproduction of her offspring. Polyandry is hypothesized to confer these fitness benefits via at least two means: females may either subsequently remate with a higher quality male to pass on “better genes” to their offspring or remate with different males to increase genetic diversity within clutches (Yasui 1998). The latter genetic diversity hypothesis posits that mating with multiple males is a female bet‐hedging strategy, employed either when females are unable to accurately gauge the quality of male partners or when the environment is unpredictable, making it impossible to select gene variants that will be beneficial in the next generation (Yasui 1998).

Polyandry also sets the stage for sperm competition, in which sperm from different males contend for fertilization within the female reproductive tract. In many invertebrate and bird species, this typically results in the vast majority of offspring being sired by the last male—a phenomenon called last male sperm precedence (LMSP) (Singh et al. 2002; Schnakenberg et al. 2014). With regards to the hypothesized indirect fitness benefits of polyandry described above, if females remate for “better genes,” LMSP benefits both females as well as their last mate, increasing both female and male's reproductive success. However, if females remate to increase offspring genetic diversity, LMSP benefits the last male mate at the cost to the female as offspring genetic diversity is reduced. In this latter hypothesis, the reproductive interests of males and females are misaligned and interlocus sexual conflict would likely arise as a result of this imbalance (Chapman 2006). Over time, selection on females may have favored the emergence of mechanisms that mitigate LMSP. However, such counteradaptations remain unknown.

Our understanding of the mechanistic underpinning of paternity allocation is unfortunately incomplete. This is likely due in part to the inherent complication of observing events concealed within the female reproductive tract. In response to this obstacle, Manier et al. (2010) generated *Drosophila melanogaster* transgenic males with red or green fluorescently labeled sperm and sequentially mated them to females to observe sperm fate. After mating, *D. melanogaster* females actively store sperm. This process requires an intact and functioning female central nervous system (Arthur et al. 1998). Sperm is stored in two different storage organs that are distinct in morphology and function (for review see Schnakenberg et al. 2014). In short, the seminal receptacle (SR) is a tubular organ containing at maximum about 400 sperm immediately accessible for fertilization; and the paired spermathecae (Sp) are mushroom‐shaped long‐term storage organs housing about 100 sperm each that will be used days following insemination (Manier et al. 2010; Pitnick et al. 1999). When females remate, newly acquired sperm enters these organs and displaces resident sperm, a process that ceases a few hours later when the female ejects the mating plug and all sperm not in storage (Manier et al. 2010).

The combined sperm displacement in the SR and the Sps establishes not only the ratio of sperm from each male in storage, but also ultimately offspring paternity as patterns of sperm storage significantly correlate with patterns of fertilization (Manier et al. 2010). Despite its impact on fitness, our current understanding of the principles governing sperm displacement is incomplete, particularly with respect to the female contribution to this process. Although displacement occurs in both sperm storage organs, the SR shows a higher rate of displacement compared to the Sp (Manier et al. 2010). One explanation is an unequal involvement of the female central nervous system governing sperm entrance into the two organ types. Indeed, previous work has demonstrated that a disrupted female central nervous system more severely limits storage in the Sp than in the SR (Arthur et al. 1998). This suggests that within a competitive context, Sps may experience lower displacement rates due to active female control; and on the other hand, the SR may have a greater rate of displacement due to low female involvement and therefore high levels of sperm competition.

The identification of factors that influence sperm displacement and patterns of paternity in *Drosophila* have come from investigations employing paradigms that have intentionally reduced variation in female mating behavior. The canonical protocol includes mating a female to two phenotypically distinct males one to five days apart (see Table S1), genotyping the resulting offspring, and expressing the paternity as a proportion: offspring sired by the first male, P1; or the second, P2 (Boorman and Parker 1976; Manier et al. 2010; Lüpold et al. 2012). Usually, studies indicate a P2 of ∼80% (see references Table S1), which can be influenced by genetic and environmental factors acting on males (for a review see Singh et al. 2002; Schnakenberg et al. 2014). These studies have undeniably advanced our understanding of principles governing the male contribution to postcopulatory sexual selection, namely sperm competition. The use of this paradigm has also led to identifying several female factors that are linked to deviations in LMSP such as female genetics (Clark and Begun 1998; Clark et al. 1999; Reinhart et al. 2015), reproductive tract morphology (Bangham et al. 2003), age (Mack et al. 2003), and developmental condition (Amitin and Pitnick 2007). However, the imposed mating behavior constraints, such as number of partners and standardized mating latency, may be masking additional female contributions to paternity allocation. Although these studies demonstrated the influence of male and females factors that influence LMSP, the role of female behavioral decisions in paternity distribution in *Drosophila* remains an open question.

Previously, the timing of sperm ejection after the second mating has been shown to correlate well with male fertilization success: longer mating‐ejection latency was associated with increased storage of second male sperm in the SR (Lüpold et al. 2013). As ejection not only precedes but is also temporally coupled with remating (Laturney et al. 2016), it is likely that variation in mating rate, previously held constant in standard polyandry paradigm, may also be associated with sperm storage and paternity outcomes. Although little is known about the remating behavior of *Drosophila* in nature (Giardina et al. 2017), it is clear that females remate often as wild‐caught females typically hold the sperm of four to five males (Milkmann and Zeitler 1974; Ochando et al. 1996; Harshman and Clark 1998; Imhof et al. 1998; Morrow et al. 2005) and various laboratory paradigms that accommodate continuous interaction between males and females observe remating within a few hours of the virginal mating (Kuijper and Morrow 2009; Billeter et al. 2012; Krupp et al. 2013; Gorter et al. 2016; Smith et al. 2017). In continuously interacting social groups, patterns of remating are mostly mediated by the female, as between strains differences in mating frequencies and temporal distribution of mating events are consistent with the genotype of the females regardless of the genetic background of the males with which they are housed (Billeter et al. 2012). Although aspects of female mating rate modulate LMSP in other arthropods (Zeh and Zeh 1994; Arnaud et al. 2001; Drnevich 2003; Blyth and Gilburn 2005) and previous studies in *Drosophila* have highlighted the potential for females to actively modulate sperm storage and/or displacement (Arthur et al. 1998; Adams and Wolfner 2007; Avila and Wolfner 2009; Chow et al. 2013; Schnakenberg et al. 2014), no study dedicated to investigating variation in remating rate in continuously interacting social groups and the resulting violations to LMSP in *D. melanogaster* has been performed.

Here, we tested whether female remating rate, defined as the number of mates and the interval between matings, can influence patterns of sperm storage and subsequent paternity in *Drosophila*. To monitor paternity of offspring and male‐specific sperm fate, we engineered three strains of transgenic male flies producing sperm fluorescing either green, red, or blue, generated in the style of Manier et al. (2010). Using these transgenic strains, we were able to visualize and quantify sperm from multiple males in the intact female reproductive tract post‐copulation. We report that thrice‐mated females that remate in quick succession produce fewer offspring and have fewer stored sperm from their most recent mate compared to either thrice‐mated with longer intervals or twice‐mated female with any interval length. Analysis of storage patterns of thrice‐mated females revealed no sperm precedence in the Sp, consistent with the finding that this storage organ has less exchange between resident and newly acquired sperm than the SR in twice‐mated female (Manier et al. 2010). In summary, we find that the number of copulations and the time interval between the last and the penultimate mating predicts the outcome of sperm competition, suggesting that females can modulate the strength of LMSP by modulating remating rate.

## Material and Methods

### *DROSOPHILA* STOCKS

Flies were reared on food medium containing agar (10 g/L), glucose (167 mM), sucrose (44 mM), yeast (35 g/L), cornmeal (15 g/L), wheat germ (10 g/L), soya flour (10 g/L), molasses (30 g/L), propionic acid and Tegosept. Flies were raised in a 12‐h light:12‐h dark cycle (LD 12:12) at 25°C. Virgins were collected using CO_2_ anesthesia and were aged in same‐sex groups of 20 in vials for five to seven days prior to testing. Females were from the wild‐type strain *Canton‐S*. Males were of the y^1^, M{vas‐int.Dm}ZH‐2A w^*^; M{3xP3‐RFP.attP}ZH‐102D (Bloomington stock number 24488) genotype with a transgenic protamine B fusion protein with one of three fluorescent markers inserted in the attP site: eGFP, mCherry, or mTurquoise referred to as green fluorescent protein (GFP), red fluorescent protein (RFP), or blue fluorescent protein (BFP), respectively. For details regarding the generation of these fluorescently tagged sperm strains see Supporting Information. Protamine B and either GFP, RFP, or BFP (Jayaramaiah Raja and Renkawitz‐Pohl 2005; Manier et al. 2010) can be easily visualized in the male testes or in the reproductive tract of a mated female (Fig. S1a, c, and d). Although green and red (but not blue) sperm had already been generated by Manier et al. (2010), we generated new versions of these transgenes that are now introduced into the same genetic background and genomic location using the PhiC31 integrase system to minimize variation between transgenic lines (Bischof et al. 2007). Wild‐type females once‐mated to transgenic males expressing one of the three fluorescent proteins did not differ in quantity of offspring produced (one‐way ANOVA, *F*(2, 48) = 1.77, *P* = 0.69; Fig. S1b). Therefore, in a non‐competitive context we find no differences in fertilization ability of the sperm indicating that the slight amino acid differences between transgenes (Hadjieconomou et al. 2011) does not impact male fecundity. Because these males have similar fecundity and are near genetically identical, there is no evidence for competitive difference, which suggests that variation in sperm competition ability between these three types of males is caused by the female.

### MATING PARADIGM

**Figure 1:**
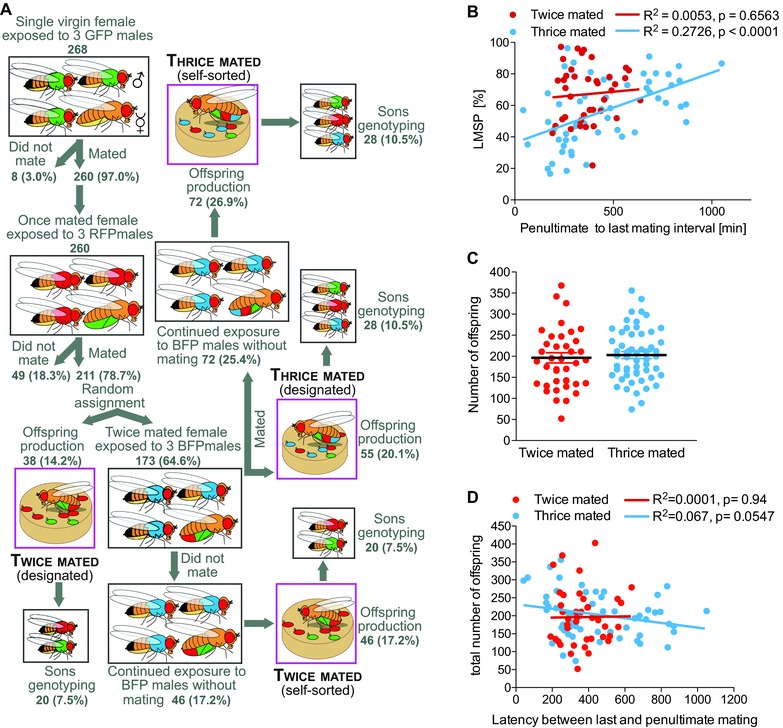
Female remating rate modulates last male sperm precedence (LMSP). (A) Cartoon representing the mating scheme. Females (numbers indicated in the figures) were sequentially exposed to two or three different types of males expressing either green, red, or blue transgenic fluorescent sperm (indicated by the color of the male). Time interval between mating was recorded. Once the female mated, she was either immediately removed or placed alone in a vial to produce offspring, or the males were removed and replaced by the next group. After the second mating, females were randomly designated to the twice‐mated group or exposed to new males. A portion (indicated in percentages in the figure) of the twice‐ and thrice‐mated females were removed from the chamber directly after mating and placed alone in a vial for progeny production (designated group), the rest remained in the mating chambers after mating two or three times, and did not have additional copulations, which are referred to as self‐sorted. The females were isolated and placed in a food vial to lay eggs until they stopped producing fertilized eggs. All their sons were then counted and subjected to paternity testing. (B) Correlation between LMSP and remating latency between last and penultimate mating (GFP and RFP males for twice‐mated females represented in red; RFP and BFP for thrice‐mated females represented in gray). Strength statistical significance of the relationship was assessed with a Pearson's correlation test. (C) Mean number of offspring produced by females who mated twice or thrice. Differences between groups were assessed with a two‐tailed Student's *t*‐test, which indicated no significant difference between the two groups; *P* = 0.639. Error bars indicate SEM. (D) Relationship between total number of offspring produced and remating latency between last and second last mating (GFP and RFP males for twice‐mated females represented in red; RFP and BFP for thrice‐mated females represented in gray). Strength statistical significance of the relationship was assessed with a Pearson's correlation indicated above the graphs.

The mating chamber consisted of a 10 × 35 mm Petri dish layered with 3 mL of food medium (as above) with the addition of 105 g/L of yeast to increase mating rate (Gorter et al. 2016). Chambers containing flies were observed for a maximum of 24 h by an observer and/or a Logitech 910C webcam in combination with Security Monitor Pro software (Deskshare, Inc., Plain View, NY) as described in Gorter and Billeter (2017). The onset time of all matings were recorded. To produce twice‐ and thrice‐mated females (Fig. 1A), single virgin females were transferred to mating chambers using a mouth pipette at Zeitgeber Time 0 (ZT0, 9 am). Three males were added to each chamber all expressing the same fluorescent marker, ProtB::GFP (GFP). After copulation was observed, males were replaced with three virgin RFP males. After a successful remating, females were randomly designated to the twice‐ or thrice‐mated condition. Twice mated (designated) are females who were immediately removed from the chamber and isolated for progeny production (Fig. 1A). Thrice mated (designated) are females who remained in the mating chamber, were exposed to three virgin BFP males, and were observed to mate with one of them (Fig. 1A). These females were immediately removed from the chamber after mating and isolated for progeny production.

To control for female factors that may affect LMSP, such as cryptic female choice related to exposure to males with whom they do not mate, we included treatment groups where twice‐ and thrice‐mated females remained in the mating chambers (<20 h) after their final mating and interacted with males but did not mate (Fig. 1A). Following the assay, females were removed from the chamber and isolated for progeny production. These females are referred to as “self‐sorted” because they had the opportunity to mate an additional time but did not.

To produce once‐mated females and explore unintended difference between males from different fluorescent marker lines, virgin females were placed in mating chamber with three males with the same fluorescent marker (green, red, or blue), immediately removed from the chamber after mating, and isolated for progeny production.

### PROGENY PRODUCTION

After the mating paradigm, females were placed individually into a fresh vial containing 10 mL of food medium. Females were transferred at least three times thereafter: 48 h (day 2), 150 h (day 6), and 216 h (day 9) after the start of the experiment (ZT0 on day 0) until they stopped fertilized eggs production. Offspring were counted, and male offspring were placed into a small vial, flash frozen with liquid nitrogen, and placed at −20°C until genotyping.

Eggs laid during the mating paradigm in the mating chamber were not systematically collected and therefore not included in the analysis. To estimate the potential weight of these eggs in the final brood of the females, we counted the number of eggs laid during the length of the assay (max 24 h) by 110 of the 268 females who were assayed. On average, females laid 10 eggs during the assay (Fig. S2). We estimate that the percentage of offspring produced during the period of the assay (taking into account that 80% of eggs develop into viable adults) is 4% and that this would not significantly alter our LMSP calculations and our conclusions.

### PATERNITY ANALYSIS OF MALE OFFSPRING

Paternity was assessed by inspecting the testes from all male offspring for the expression of either GFP, RFP, or BFP‐sperm using a Leica MZ10F fluorescence stereomicroscope equipped with filters to visualize the different fluorescent signals. As the sex ratio of offspring produced from mating females with a male expressing either of the three fluorescent sperm types was equal (1:1.05 male/female brood tested = 16–18 per male genotype), genotyping sons of a multiple‐mated females acts as a good proxy for the general pattern of offspring paternity.

Of the 268 females who went through the mating paradigm, we randomly selected 20 females from the twice‐mated designated and 20 females from the twice‐mated self‐sorted as well as 28 females from the thrice‐mated designated and 28 females from the thrice‐mated self‐sorted, for paternity testing of all their sons. Females who died during progeny production were excluded so as to guarantee a full account of a female's brood. We also excluded females that did not produce offspring from all two or three males they were recorded to mate with to exclude females who failed to receive ejaculates from one of their mates and hence were pseudomated.

### IMAGING OF THE THRICE‐MATED FEMALE REPRODUCTIVE TRACT

We imaged the reproductive tracts of 31 thrice‐mated females. Females were mated according to the mating paradigm. However, after the third mating females remained in the mating chamber until they ejected sperm from the last male, which marks the completion of the process of sperm storage (Manier et al. 2010). Once ejected, females were placed individually into 1.5 mL Eppendorf tube, flash frozen with liquid nitrogen, and placed at −20°C until processing. The reproductive tract of each female was removed, mounted onto a glass slide with Vectashield (Vector Laboratories, Burlingame, CA), and a coverslip placed on top. Samples were imaged with Leica TCS SP8 confocal microscope with a 40× oil immersion lens. Sperm with fluorescent tags were manually counted in the different optical slices using FIJI software with the cell counter plugin.

### STATISTICAL ANALYSIS

A standard multiple regression model was performed in SPSS (IBM Corp., Armonk, NY) to determine if number of copulations, method of group assignment (designated or self‐sorted), and last to penultimate remating latency significantly influenced LMSP. Preliminary analyses were conducted to ensure no violation of the assumptions were committed. All predictors had a moderate and significant correlation with the dependent variable; collinearity diagnostics was performed and both tolerance and variance inflation factor were in an acceptable range (greater than 0.1 and less than 10, respectively); and the distribution of the data were visually assessed with a scatterplot. No violations of the assumptions were found. All other statistical analysis was performed using GraphPad Prism 5 (GraphPad Software, Inc., La Jolla, CA). These datasets were first analyzed with a Kolmogorov–Smirnov test (with Dallal–Wilkinson–Lillie for *P*‐value) to test for normality, which was satisfied in all cases.

## Results

### LMSP IS REDUCED IN THRICE‐MATED FEMALES

Twice‐ and thrice‐mated females were generated according to the mating paradigm described in Figure 1A. This resulted in the quantification of the remating intervals and progeny of 96 females randomly chosen out of the 268 females assayed and the paternity analysis of over 9000 of their sons (see Methods). We determined the mean percentage of offspring sired by the first male (P1) and second male (P2), and in the case of a third mating, the third male (P3). This allowed comparing variation in female's remating rate (number of mates and remating latency) and its correlation with LMSP.

To examine if number of mates and remating latency impacts LMSP, we performed a standard multiple regression. In addition to this, we also explored if group assignment method (designated or self‐sorted; see Methods and Fig. 1A) contributed to paternity patterns. The total variance explained by the model as a whole was 24.1% (*F*(3, 92) = 9.742, *P* < 0.001). Two of the factors had a statistically significant effect on LMSP: number of female copulations (beta = −0.324, *P* = 0.001) and female remating latency (beta = 0.297, *P* = 0.015). The method of group assignment was not a significant predictor (beta = 0.165, *P* = 0.164). Therefore, female remating rate (number of mates and time between matings) is the main factor that influenced LMSP in this experimental design. As group assignment did not significantly influence LMSP, all twice‐ and thrice‐mated females were grouped together for analysis.

To further investigate the relationship between female remating behavior and paternity, we assessed the strength of the association between timing of remating and LMSP in both twice‐ and thrice‐mated females. We found a significant correlation between remating latency and proportion of offspring sired by the last male in thrice‐mated females, but failed to find this relationship in twice‐mated females (Fig. 1B). We conclude that when females mate with three males, the proportion of offspring sired by the last male is maximized when the copulation latency is increased and is minimized when the copulation latency is decreased.

We also investigated the total number of offspring produced by twice‐ and thrice‐mated females and at different intervals to determine the effect of remating rate on fecundity. Neither the number of matings (Fig. 1C) nor the time between these matings (Fig. 1D) had any significant effect on total number of offspring produced. This suggests that unlike LMSP, female fecundity is not influenced by female mating rate in our experimental design.

Taken together, these data indicate that female remating rate modulates LMSP without altering female fecundity. This suggests that increasing female mating behavior allows for greater clutch genetic diversity because the progeny share of offspring produced by thrice‐mated females with short remating latencies was more evenly distributed between the sires.

### REMATING RATE AFFECTS SPERM STORAGE IN THE FEMALE SEMINAL RECEPTACLE

**Figure 2:**
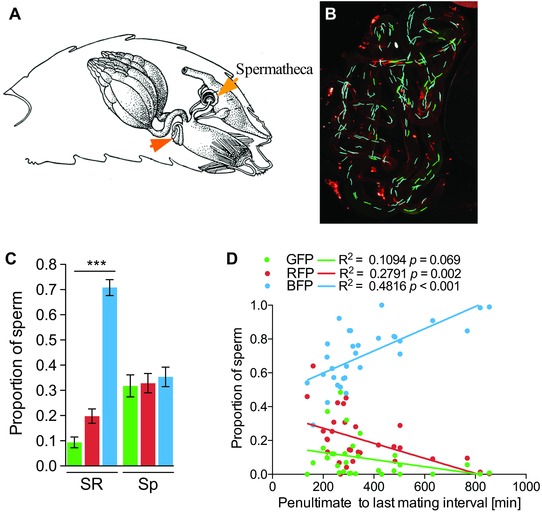
Female remating rate modulates sperm storage. (A) Cartoon representing a female abdomen with the reproductive organ in situ. The location of the seminal receptacle (SR) and spermathecae are indicated by arrow. Cartoon modified from Miller (1950). (B) Confocal microscopy micrograph of the SR of a thrice‐mated female holding green, red, and blue fluorescent sperm. (C) Portion of green, red, and blue sperm in either the SR or in the spermathecae (Sp) of a thrice‐mated female. Error bars indicate SEM number of replicates. (D) Correlation between proportion of green, red, or blue sperm in storage in the SR and remating latency between penultimate and last mating in thrice‐mated females. Strength and statistical significance of the relationship was assessed with a Pearson's correlation.

Because previous findings have established a high association between patterns of paternity and ratios of sperm storage in twice‐mated *D. melanogaster* females (Manier et al. 2010), it is likely that differences in the patterns of paternity produced by twice‐ and thrice‐mated females in the present study is a direct reflection of sperm storage in the female sperm storage organs (illustrated in Fig. 2A). Therefore, the relationship between remating latency and LMSP we found in thrice‐mated females, but failed to reveal in twice‐mated females, is likely due to altered sperm displacement: the exchange between resident and newly acquired sperm within the female sperm storage organs.

To test this hypothesis, we quantified the proportion of stored sperm expressing the green, red, or blue fluorescent tag (Fig. 2B) in both Sp and SR of thrice‐mated females (Fig. 2C). We found that females stored a larger fraction of sperm from the last male (BFP‐labeled sperm) compared to both second and first males (RFP‐ and GFP‐labeled, respectively) in their SR (short‐term storage; KW = 62.59, *P* < 0.0001), but had equal amounts of sperm from all males stored in the Sp (long‐term storage; one‐way ANOVA *F* (2, 90) = 0.21, *P* = 0.81) (Fig. 2C). These data indicate no LMSP, and thus no mate order effects, in the Sp in thrice‐mated females, but strong sperm precedence in the SR.

As remating latency was significantly correlated with P3 (Fig. 1B), we predicted a relationship between remating latency and portion of sperm from all three males in this organ. We correlated the fraction of sperm from the first (GFP), second (RFP), and last male (BFP) with time interval between the last and penultimate mating in thrice‐mated females (Fig. 2D). As predicted, the fraction of sperm from the last male (BFP) in the SR was significantly correlated with remating latency: females that remated faster had fewer BFP‐labeled sperm compared to females with longer remating latencies (Fig. 2D).

Taken together, thrice‐mated females with an increased remating rate exhibit reduced LMSP by storing more equal ratios of sperm from all males within the SR. These results confirm our findings using paternity assessment that female mating rate modulates sperm precedence (Fig. 1B) and extend them to suggest a mating rate‐dependent modulation in sperm storage specifically happening in the SR and not the Sp.

## Discussion

One proposed explanation of polyandry is its potential to increase the reproductive success of females via expansion of offspring genetic diversity (Yasui 1998; Jennions and Petrie 2000; Parker and Birkhead 2013). However, the widespread phenomenon of LMSP, strongly biasing paternity in favor of the last male, limits the scope for such genetic benefits. In response to this, sexual conflict theory predicts that selection should favor female mechanisms that reduce males’ ability to manipulate paternity. Here, we show that *D. melanogaster* females who remate in quick succession with three males counteract LMSP, maintaining a more evenly distributed paternity and thereby increasing the genetic diversity of their offspring. Thus, modulation of paternity via remating behavior may have evolved as a counteradaptation to male traits that promote LMSP.

Most research on LMSP in this species has focused on a two‐male competitive assay with at least one 24‐h interval of isolation between matings. Because *D. melanogaster* females are documented to mate with several males in the wild, we investigated whether lessons learnt from the two‐male scenario extend to a perhaps more natural situation when females mate with more males, more frequently. Despite the modifications to the paradigm, we still observed LMSP in thrice‐mated females, but it was less strong than in twice‐mated females. We also observed next to no sperm displacement in the Sp in comparison to the SR as detected with the previous experimental design (Manier et al. 2010). Together, these results suggest that the mechanism of sperm displacement in the Sps is biased by neither mating latency nor mating order, allowing for Sps to provide equal storage for all mates. Most of LMSP therefore happens in the SR. In addition to supporting previous findings, we were also able to highlight the importance of variability of female reproductive behavior. By regulating mating rate, previous research has identified important female‐derived factors of postcopulatory sexual selection. However, by standardizing female mating behavior, previous paradigms have also simultaneously abolished meaningful consequences of plasticity in this trait. In contrast to the previous paradigm, we accommodated for variation in female mating rate and extended our understanding of the impact of female mating behavior on offspring production. By utilizing this new approach, we revealed that the duration between matings is a critical element in the outcome of paternity.

Uncovering the factors that influence female mating behavior will allow researchers to address such fundamental questions about the extent of female control over reproduction. More specifically, the relationship between remating rate and LMSP in *D. melanogaster* uncovered here demonstrates a key entry point into the cellular and molecular underpinning of postcopulatory sexual selection. As both mating and sperm storage are active processes (Arthur et al. 1998; Zhou et al. 2014; Aranha et al. 2017), there is much potential to use this genetic model to gain access into the neuronal architecture of female control over paternity. Moreover, the propensity of *D. melanogaster* females to remate is not only influenced by food availability (Gorter et al. 2016), current nutritional status (Fricke et al. 2010), and developmental conditions (Amitin and Pitnick 2007), but it has also been linked to natural female genetic variation (Arthur et al. 1998; Giardina et al. 2011; Billeter et al. 2012). Remating rate also increases with group size (Gorter et al. 2016) and group genetic diversity (Krupp et al. 2008; Billeter et al. 2012), suggesting that females can detect variation in her social group and adapt her mating rate to maximize her reproductive success. We acknowledge that male–male competition likely also has an impact on LMSP in thrice‐mated females as male‐derived seminal fluid peptides within the male ejaculate can affect sperm storage, female sexual receptivity, and fecundity. Moreover, the transfer of these peptides can be modulated based on the perception of the female mating status (Wigby et al., 2009; Sirot et al. 2011; Wigby et al., 2016). These factors were not explored in our study, but their impact was mitigated by the usage of genetically similar males. Moreover, the fact that the female, and not male, genotype is the main factor influencing remating intervals in the mating assay used here (Billeter et al. 2012) indicates that females are in control of their remating rate, perhaps as a means of protecting genetic diversity in their clutches, as demonstrated here.

The correlation between the timing of remating and paternity patterns uncovered here allows us to speculate on the potential mechanisms that achieve the modulation of LMSP. Because we observe reduced LMSP associated with short remating latencies, it is possible that resident sperm's defensive ability decreases over time. Therefore, the timing of the exposure of the resident sperm to the newly acquired ejaculate may influence sperm competition outcomes. Additionally, other postcopulatory events independent of remating latency may also influence the displacement process such as the timing of sperm ejection (Lüpold et al. 2013). Following remating, recently acquired sperm displaces the resident sperm from the sperm storage organs until the female removes the unstored ejaculate via ejection (Manier et al. 2010). The longer the process continues, the more exchange can occur, resulting in increased LMSP (Lüpold et al. 2013). Moreover, we have shown that females remate shortly after sperm ejection (Laturney et al. 2016), which suggests that females who are quick to remate are also likely quick to eject, offering prospective support for ejection as the potential mechanism governing the magnitude of the displacement process. Although previous reports on the effect of sperm ejection on LMSP focused on twice‐mated females, isolated between matings, with remating latencies between two and four days, it is likely that a similar mechanism may also influence the outcome of sperm competition within the experiment context employed in this present investigation.

Polyandry has been observed in females of various species ranging from insects to marsupials (Kraaijeveld‐Smit et al. 2002; Friesen et al. 2014; Rovelli et al. 2015). More intriguing, the relationship between female mating latency and paternity allocation has been observed in multiple species (Zeh and Zeh 1994; Arnaud et al. 2001; Blyth and Gilburn 2005; Drnevich 2003, for a review see Simmons 2001). This demonstrates that across taxa irrespective of the species‐specific biochemistry, genetic architecture, and physiology, females who remate more often produce more equal paternity shares, suggesting that not only is female remating behavior plastic, but also that females of various species may have evolved the same adaptation to combat paternity manipulation.

As new technologies allow for greater inspection into the principles governing sexual reproduction, we gain greater insight into how the genetic makeup of the next generation is determined and the explicit role that a force such as conflict theory plays. This present study highlights that offspring genetic diversity depends on the number of mates a female acquires as well as the timing of those matings. If paternity confers drastically different chances of survival and/or reproduction to the offspring, then not only the “who,” but also the “when” of female mating behavior have important evolutionary consequences.

## Conclusion

Our results provide further support to a growing body of evidence demonstrating that females exert postcopulatory sexual selection (Firman et al. 2017). Similar to other insects, arthropods, and mammals, aspects of female remating behavior such as remating rate, a combination of remating latency, and number of sexual partners, modulate LMSP (Zeh and Zeh 1994; Drnevich 2003) and therefore variation in polyandry results in different patterns of paternity in *Drosophila*. By modulating the paternity, females can maximize benefits of polyandry and increase offspring genetic diversity. These findings also suggest that there may be active control over the sperm storage process by the female and hint that mechanisms of cryptic female choice are at play.

Associate Editor: Dr. R. Snook

## Supporting information


**Table S1**. Survey of the experimental design used to investigate last male sperm precedence.
**Figure 1**. Transgenic males expressing fluorescent protein‐labeled sperm heads.
**Figure 2**. Number of eggs laid by females in the mating chamber.
