# Supplementary material for: Last male sperm precedence is modulated by female remating rate in Drosophila melanogaster
Source: Evol Lett. 2018 Apr 13;2(3):180–9. doi: 10.1002/evl3.50 (PMC6121866; doi:10.1002/evl3.50)
Supplement: Supplementary file 1 — Table S1. Survey of the experimental design used to investigate last male sperm precedence. Figure 1. Transgenic males expressing fluorescent protein‐labeled sperm heads. Figure 2. Number of eggs laid by females in the mating chamber. [file EVL3-2-180-s001.pdf]

Supplementary material

*Fluorescent marker donor vectors*

*Nde*I restriction sites were introduced at the 5'- and 3'-ends of fluorescent markers eGFP, mTurquoise and mCherry by amplification with primers *Nde*GFP ('5-tgcatatgcaaggtgagcaagggcgaggagctgttcacc-3') and pKC26fB2,0.Fp-rev (5'-gcccatatgttacttgtacagctcgtccatgc-3') for eGFP and mTurquoise and primers *Nde*mCherry (5'-tgcatatgcaaggtgagcaagggcgcggaggataacat-3') and pKC26fB2,0.Fp-rev for mCherry. Fragments were ligated into pJET1.2 vector (Thermo Scientific) by blunt-end ligation and cloned XL1-blue colonies were screened by restriction analyses.

*Protamine B::fluorescent marker CDS*

In order to create a CDS for Protamine B fusion protein with integrated eGFP, mTurquoise of mCherry, fluorescent markers were excised from the subcloned pJET1.2 plasmids using *Nde*I and ligated into the pBS/ProtB4.2KP*Nde*I (Manier et al., 2010) vector. Fragments were integrated in frame with the CDS on position 45 of exon 3 of the ProtB ORF via an introduced *Nde*I restriction site. Orientation of the fluorescent fragments (3' to 5') in the pBS/ProtB4.2KP-NdeI vector were determined by restriction analysis using *Dpn*I.

*Fluorescent protamine B cloning and expression vector*

To create vectors for generating *Drosophila* expressing Protamine B::fluorescent marker fusion proteins, 4.8Kb *Xba*I fragments of the different pBS/ProtB4.2KP constructs containing the introduced florescent markers were subcloned into the *Xba*I side of plasmid pUAST-attB1. The vector used in this study contains an inactivated 5x UAS-hsp70 site, obtained after digestion with *Pst*I and religation of the 8.2Kb fragment. Orientation of the

inserted protamineB::fluorescent marker CDS was determined by restriction analysis with BamHI. Sequence analysis of the reading frame of fluorescent markers and conformation of the direction of the protamineB::fluorescent marker CDS in pUAST-attB-ProtB::eGFP and pUAST-attB-ProtB::mTurquoise was performed using the primers Seq-ProtB-for (5'-ggacctgtcactaacaac-3'), Seq-GFCITU-rev (5'-gatgttgtggcggatctt-3') and SV40poly(A).1-rev (5'caccacagaagtaaggttcct) and primers Seq-ProtB-for, Seq-ProtB-rev (5'-gcgctattccaacatccta-3') and SV40poly(A).1-rev for pUAST-attB-ProtB::mCherry.

*Generation of transgenic Drosophila*

To generate transgenic *Drosophila* with eGFP, mTurquoise or mCherry expressing sperm heads, the different pUAST-attB vectors with protamine B::fluorescent marker CDS were used to inject PhiC31-containing attP docking site embryos of *Drosophila melanogaster* with genotype y[1] M{vas-int.Dm}ZH-2A w[*]; M{3xP3-RFP.attP}ZH-102D (BDSC stock number 24488). Embryo injection and generation of stable transformants was performed at BestGene Inc., U.S.A.

| | 1st mating | | | | Interval | | 2nd mating | | | | Offspring | |
|---|---|---|---|---|---|---|---|---|---|---|---|---|
| | males | female | Dur | Var | Isolated | Dur | males | female | Dur | Proof | Genotyping | Dur |
| Boorman and Parker 1976 | 1 | 1 | NS | Ob | yes | 4-14 days | 1 | 1 | 5 | Ob | irradiation treatment | life-time |
| Lefevre and Jonnson 1962 | 1 | 1 | NS | Ob | no | 0-24 hrs | 1 | 1 | ns | Ob/Off | mutant, forked | life-time |
| Hughs 1997a | 1 | 2 | 2 | Off | yes | 3 days | 1 | 2 | 24 | Off | mutant, eye colour | 10 |
| Hughs 1997b | en masse | en masse | NS | Off | yes | 3 days | 1 | 2 | 24 | Off | mutant, eye colour | 10 |
| Clark et al. 1995 | en masse | en masse | 2 | Off | yes | 2,5 days | 1 | 3 | 12 | Off | mutant, eye colour | 13 |
| Prout and Bundgaard 1977 | 100 | 100 | 2 | off | yes | 36 | 1 | 3 | 24 | Off | mutant, eye colour | 12 |
| Clark et al. 1998 | en masse | en masse | 2 | Off | yes | 2 days | 1 | 2 or 3 | 18 | Off | mutant, eye colour | 13 |
| Gilbert and Richmond 1981a | 1 | 1 | NS | Ob | yes | daily | 1 | 1 | 2 | Ob | mutant, forked | 7 |
| Gilbert and Richmond 1981b | varied | varied | 7 days | Off | no | none | NA | NA | NA | NA | mutant, forked | 9 |
| Gilbert and Richmond 1981c | 1 | 1 | 24 | Off | no | 0 | 1 | 1 | 24 | Off | mutant, forked/eye-colour | 8 |
| Morrow et al. 2005 | 16 | 24 | 1,5 | Off | no | (0-24) | 16 | 16 | 24 | Off | mutant | <24 hrs |
| Chapman et al. 2000a | 6 | 7 to 9 | NS | Ob | yes | 2 day | 2 | 1 | 18 | Off | mutant, eye colour | 13 |
| Chapman et al. 2000b | 1 | 1 | NS | Ob | yes | 3-5 days | 1 | 1 | NS | Ob | irradiation treatment | lifetime |
| Chapman et al. 2000c | 6 | 7 to 9 | NS | Ob | yes | 1-2 day | 2 | 1 | 18 | Off | mutant, eye colour | 13 |
| Scott and Richmond 1990 | 1 or 2 | 1 | NS | Ob | yes | 6 hrs | 1 | 1 | 1 | Ob | yellow body, attached X | 6 |
| Harshman and Prout 1994a | 20 | 20 | 24 | Off | yes | 4 days | 20 | 20 | 48 | Off | mutant, eye colour | 20 |
| Harshman and Prout 1994b | 20 | 20 | 24 | Off | yes | 4-6 days | 2 | 1 | 3 | Ob | used sterile males | 20 |
| Price et al. 1999 | 1 | 1 | 10 | Ob | yes | 1 day | 1 | 1 | 10 | Ob | mutant, eye colour | life-time |
| Price 1997 | 1 | 1 | 10 | Ob | yes | 1 day | 1 | 1 | 10 | Ob | mutant, eye colour | life-time |
| Civetta and Finn 2014 | 1 | 1 | 8 | Ob | yes | 2 days | 1 | 1 | 8 | Ob | mutant, body colour | life-time |
| Manier et al. 2010 | 1 | 1 | NS | Ob | yes | 3 days | 1 | 1 | 6 | Ob | fluorescent protein | varied |
| Clark and Begun 1998 | en masse | en masse | 2 | Off | yes | 2 days | 2 or 3 | 1 | 18 | Off | mutant, eye colour | 11 |
| Civetta and Clark 2000 | en masse | en masse | 2 | Off | yes | 2,5 days | 1 | 3 | 12 | Off | mutant, eye colour | 13 |
| Clark et al 1999 | en masse | en masse | 2 | Off | yes | 2 days | 2 or 3 | 1 | 18 | Off | mutant, eye colour | 11 |
| Ala-Honkola et al. 2014 | NS | NS | NS | Off | NS | 2-5 days | 1 | 1 | 4 | Ob | fluorescent protein | 6 |

| | | | | | | | | | | | | |
|---|---|---|---|---|---|---|---|---|---|---|---|---|
| Billeter et al. 2012 | 6 | 6 | 8 | Ob | no | none | 6 | 6 | 8 | Ob | cuticular pigment pattern | life-time |
| Bjork et al. 2007 | 1 | 1 | NS | Ob | yes | 3-4 days | 1 | 1 | NS | Ob | mutant, eye colour | 11 |
| Amitin and Pitnick 2007 | 1 | 1 | NS | Ob | yes | 2-8 days | 1 | 1 | 5 | Ob | mutant, eye colour | 1 |
| Mack et al. 2003 | 40 | 65 | 3 | Off | yes | 3 days | 2 | 1 | 18 | Off | mutant, eye colour | 12 |
| Bangham et al. 2003 | 1 | 1 | 6 | Ob | yes | 3 days | 1 | 1 | 9 | Ob | mutant, eye colour | life-time |
| Reinhart et al. 2015 | 1 | 1 | 6 | Ob | yes | 3 days | 2 | 1 | 6 or 12 | Ob/Off | mutant, eye colour | 2 or 3 |

**Supplementary Table S1. Survey of the experimental design used to investigate last male sperm precedence.** Publications listed with a lower case letter indicate multiple experiments within the same report. Dur = duration, Var = method of verification of mating, Ob = observed, Off= offspring

## Supplementary Figure 1

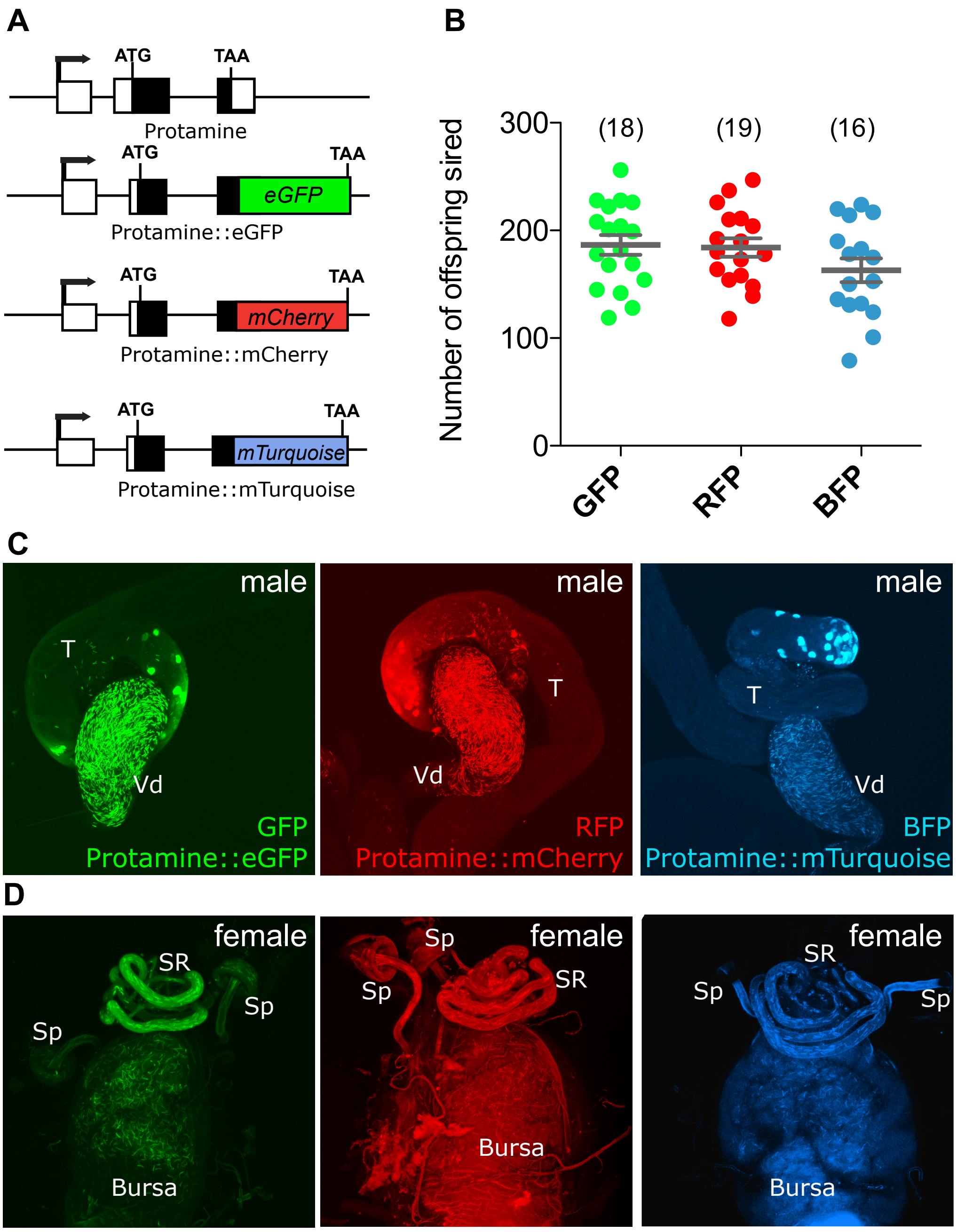


**Supplementary Figure 1. Transgenic males expressing fluorescent protein labeled sperm heads**. ***(a)*** Representation of genetic constructs derived from the ProtamineB gene. Arrow indicates the start site of transcription; ATG and TAA the Open Reading frame; boxes indicate exons. ***(b)*** Mean number of offspring produced by once-mated females mated to one of the three transgenic males expressing fluorescent protein labeled sperm; green fluorescent protein (GFP), red fluorescent protein (RFP, specifically mCherry), blue fluorescent protein (BFP, specifically mTurquoise). Differences between groups were assessed with a One-way ANOVA ($F(2, 48) = 1.767$, $p = 0.18$). Error bars indicate s.e.m. Number of replicates is between brackets. ***(c)*** Micrographs of male testes expressing fluorescent protein labeled sperm heads: GFP, RFP, and BFP, respectively. Location of the testes (T) and vas deferens (Vd), are indicated. Images are maximum projections of confocal stacks. ***(d)*** Micrograph of female sperm storage organs and anterior uterus of wild-type female mated to one of the three transgenic males: GFP, RFP, and BFP, respectively. Location of seminal receptacle (SR), paired spermathecae (Sp); and uterus/bursa (Bursa) are indicated.

# Supplementary Figure 2

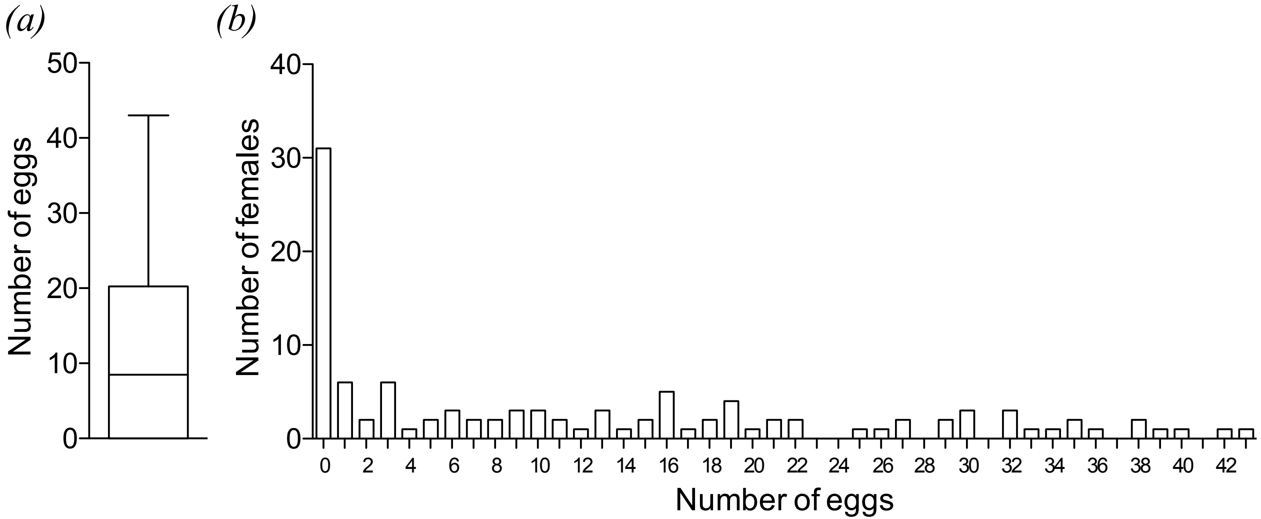


**Supplementary figure 2.** Number of eggs laid by females in the mating chamber. A) Box plot graph of the numbers of eggs laid during the assay. Box represents the interquartile range, the segment inside the box indicates the median, and the wisker above shows the maximum range. B) Histogram of the number of eggs laid by females in the mating chamber. n = 110.
